# Electronic and magnetic properties of H-terminated graphene nanoribbons deposited on the topological insulator Sb_2_Te_3_

**DOI:** 10.1038/srep29009

**Published:** 2016-07-11

**Authors:** Wei Zhang, Farideh Hajiheidari, Yan Li, Riccardo Mazzarello

**Affiliations:** 1 Center for Advancing Materials Performance from the Nanoscale, State Key Laboratory for Mechanical Behavior of Materials, Xi’an Jiaotong University, Xi’an 710049, P.R. China; 2 Institute for Theoretical Solid State Physics, RWTH Aachen University, D-52074 Aachen, Germany; 3 Institute of Energy and Climate Research (IEK-6), Forschungszentrum Jülich, D-52425 Jülich, Germany; 4 JARA-FIT and JARA-HPC, RWTH Aachen University, D-52074 Aachen, Germany

## Abstract

Magnetism in zigzag graphene nanoribbons (GNRs) has received enormous attention recently, due to the one-dimensional nature of this phenomenon, as well as its potential applications in the field of spintronics. In this work, we present a density functional theory (DFT) investigation of H-passivated GNRs on the (111) surface of the topological insulator Sb_2_Te_3_. We show that the chemical interaction between the GNR and the substrate is weak. As a result, the GNR-surface distance is large, of the order of 3.4 Angstrom, doping effects are almost negligible, and the mean-field magnetic properties of the GNR are preserved. Nevertheless, the presence of the substrate affects significantly the magnitude of the exchange coupling constants between the edges. Although our DFT calculations do not properly describe quantum fluctuations that destabilize the edge magnetism in free-standing GNRs, they provide important information about the stabilizing mechanisms which originate from the substrate-induced spin orbit coupling and the decoherence effects due to the surface states of Sb_2_Te_3_. We argue that, owing to these mechanisms, Sb_2_Te_3_ may be a suitable substrate to investigate experimentally the transition from “quantum” to “classical” magnetism in GNRs.

Two-dimensional graphene^1^ has been the focus of intensive investigation since its discovery in 2004, due to its peculiar electronic and transport properties^2^. In an effort to induce a sizable energy gap in the band structure of graphene, which could lead to ground-breaking applications in information technology, quasi one-dimensional graphene nanoribbons (GNRs) have been thoroughly investigated recently^3^,^4^,^5^,^6^,^7^. Remarkably, zigzag-terminated GNRs have been predicted to possess magnetic electronic states localized at the edge, with antiferromagnetic (AFM) coupling between the two edges^8^,^9^. Experimental evidence, albeit indirect, for the presence of edge magnetism in GNRs has been recently provided^10^. However, edge magnetism in isolated GNRs is generally not stable, in principle. There are two intrinsic sources of instability due to the one-dimensional nature of this phenomenon: quantum fluctuations brought about by the AFM inter-edge coupling, which lead to an entangled singlet ground state^11^, and thermal fluctuations^12^. The two effects can be suppressed by increasing the width of the GNR and by reducing temperature, respectively.

A generic mechanism capable of stabilizing edge magnetism is the anisotropy due to spin-orbit coupling (SOC), i.e., the dependence of the energy of electrons on the absolute orientation of their spins. A sufficiently strong magnetic anisotropy would a) damp out spin waves, leaving only domain-wall-like excitations at finite temperature, as well as b) suppress quantum fluctuations. Although the intrinsic SOC in GNRs is extremely weak, anisotropy effects can be greatly enhanced by depositing graphene on a substrate with strong SOC.

A more subtle stabilizing mechanism could occur in the presence of a substrate which possesses surface states near the Fermi energy. Such states could lead to decoherence effects and to the emergence of “classical” edge magnetism from the highly entangled singlet ground state^11^.

The last two mechanisms may be prominent in the recently discovered class of band insulators called three-dimensional topological insulators (TIs)^13^,^14^,^15^,^16^,^17^. TIs have several remarkable properties, which stem from time-reversal symmetry and strong SOC. They possess robust, conducting surface states in the bulk band gap, which exhibit spin-momentum locking and are symmetry protected against non-magnetic disorder.

Besides the stabilizing effects of the TI substrate, another interesting phenomenon is the possible back action of edge magnetism (if stable) on the TI surface state. The proximity of a TI to a magnetic system breaking time-reversal symmetry can lead to fascinating phenomena, such as an anomalous quantum Hall effect and the formation of one-dimensional chiral states induced by domain walls in the magnet^18^. Recent efforts to break time-reversal symmetry in TIs have focused on (a) depositing magnetic impurities^19^,^20^,^21^ or magnetic insulators^22^,^23^ on the surface of TIs or (b) doping TIs with magnetic impurities^24^,^25^. These proximity effects could be exploited in spintronics devices integrating TIs with magnetic materials. Since the magnetic edge states have AFM ordering across the GNR, their effect on a TI could bear some similarities with that of a domain wall.

In this work, we present a DFT study of the structural, electronic and magnetic properties of mono-hydrogenated zigzag GNRs deposited on the (111) surface of the TI Sb_2_Te_3_^26^. In principle, standard spin-polarized DFT calculations are not suitable to investigate quantum fluctuations in GNRs, because they break spin-rotational invariance from the outset. Nevertheless, they provide valuable information about the “mean-field” ground-state properties of the system, from which one can extract the substrate-induced effects on the SOC parameters and the exchange coupling constants in GNRs. Furthermore, they enable one to determine the strength of the interaction between the GNR and the TI electronic states.

Graphene-TI heterostructures have recently become an active field of research, see e.g. Refs 27, 28, 29, 30, 31. The main focus of these works was the enhancement of the spin-orbit gap in graphene due to the TI, which could open the way to experimental observation of the quantum spin Hall effect in graphene^32^. In this work, we instead investigate the effect of the TI on the edge magnetism of GNRs.

It is of great interest to study the interaction between GNRs and a substrate like Sb_2_Te_3_, which has surface states with *p* character. Recently, we have shown that H-terminated GNRs interact very strongly with metal surfaces like Ir(111), which possess *d*-like surface states near the K point of the surface Brillouin zone^33^,^34^. On the other hand, the chemical interaction with Au(111) is weak, such that the magnetic properties of the GNR are preserved^35^. The Cu(111) surface exhibits intermediate behaviour^35^,^36^. However, metal substrates are less suitable to investigate theoretically decoherence effects, in that they possess bulk bands crossing the Fermi energy, which makes the derivation of effective model Hamiltonians very cumbersome.

## Results

**Figure 1:**
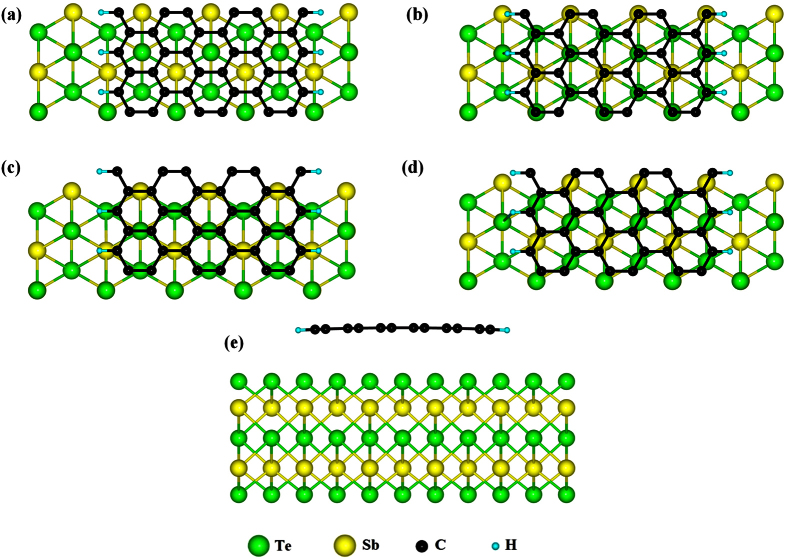
(**a**–**d**) Top view of the four adsorption configurations considered in this work. The supercell contains 3 GNR units, and the GNR has a width of 6 graphene units. (**e**) Side view of the energetically most stable adsorption configuration (shown in (**a**)). Upon relaxation, the GNR is slightly bent. Sb, Te, C, and H atoms are rendered with yellow, green, black and blue spheres.

We focus on the (111) surface of Sb_2_Te_3_ and we assume that the GNR is parallel to the (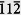) direction of the substrate. We consider Sb_2_Te_3_ as TI substrate for two reasons. First, it possesses a simple Dirac cone surface spectrum; second, the lattice constant of its (111) surface is exactly 3 times as large as that of graphene (4.26 Å versus 1.42 Å). The second property allows us to use a small supercell lattice vector along the direction parallel to the GNR (in spite of this, our models are very large and demanding from the computational point of view, as further discussed in the Methods section). We study several adsorption configurations. Top views of these configurations are shown in Fig. 1(a–d). Upon structural optimization, the most stable configuration is the one where the surface Te atoms are at the center of graphene hexagon rings (Fig. 1(a)). Notice that this is the stable structure for perfect graphene on Sb_2_Te_3_ as well^27^. The bending of the GNR is small and the distance between the GNR and the substrate is around 3.4 Å, which is also compatible with the previous study on perfect graphene^27^. The side view of the most stable configuration is shown in Fig. 1(e) and geometrical data about the relaxed GNR are shown in Table 1.

**Figure 2:**
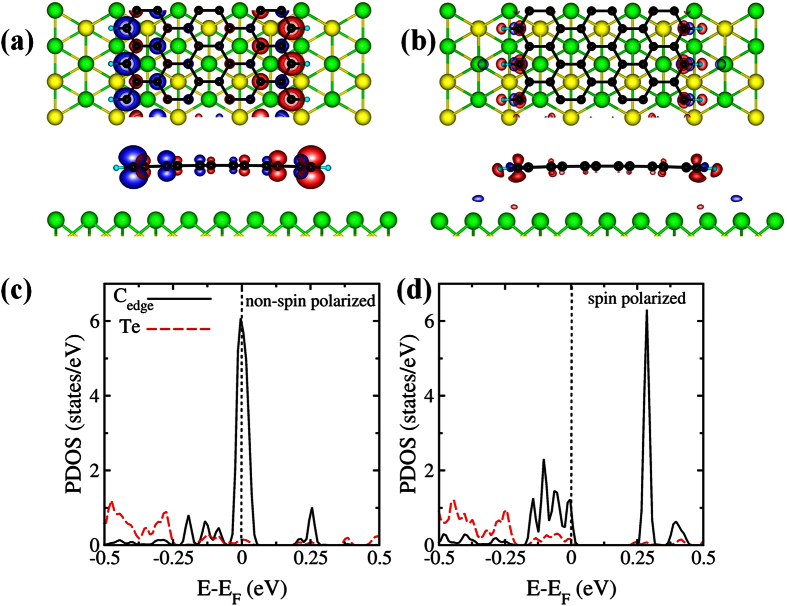
(**a**) Top and side view of an isovalue surface of the edge state spin density of the deposited GNR. The system exhibits AFM coupling between the two edges. The red (blue) surface indicates spin up (down) density. (**b**) Top and side view of two isovalue surfaces (corresponding to the values ± 5 · 10^−4 ^a.u.) of the difference between the total charge of the GNR plus substrate system and the charge of the isolated GNR and Sb_2_Te_3_. The red (blue) color indicates accumulation (depletion) of charge. (**c**,**d**) Non-spin-polarized and spin-polarized projected density of states (PDOS) for the 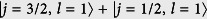 spin-angle functions of an edge C atom and a nearest neighbour Te atom. The peak at the Fermi energy in (**c**) corresponds to the edge state. The PDOS in (**d**) were calculated for spin-polarization along *x* but the direction of the polarization has negligible effect on the PDOS.

It turns out that the magnetic properties of the GNR are not strongly affected by the presence of the surface (see Fig. 2(a)). The GNR displays edge magnetism with AFM coupling between the two edges. The magnetization per edge C atom is 0.30 μ_B_/cell, which is similar to the one displayed by a free-standing GNR (using an equivalent k-point mesh). Nevertheless, the energy difference between the inter-edge ferromagnetic (FM) and AFM configuration, E_FM_-E_AFM_, is reduced significantly with respect to the free-standing case (3.9 meV versus 6.5 meV per edge C atom), due to screening effects by the surface states. This decrease in the inter-edge AFM coupling constant is beneficial for the stabilization of edge magnetism (see Discussion section).

We calculate the interaction energy between the GNR and the substrate by computing the difference between the energy of the full system and those of the isolated GNR and the clean substrate. This energy is equal to 41 meV per edge C atom, indicating weak chemical interaction between the two systems. Figure 2(b) shows that the charge redistribution upon deposition of the GNR, obtained by subtracting the total charge of the isolated GNR and substrate from the charge of the GNR plus Sb_2_Te_3_ system, is small as well. In the following, we assume that the surface is perpendicular to the *z* axis and the GNR is parallel to the *y* axis. Our calculations indicate that the easy axis of magnetization is in-plane and perpendicular to the GNR (*x*-axis). However, the magnetic anisotropy energies (MAEs) are quite small: the energy difference between the *x*-axis and the *y*-axis (respectively *z*-axis) configuration is of the order of 0.2 meV (resp. 0.1 meV). It is important to stress that these numbers should be considered as order-of-magnitude estimates of the MAEs, rather than exact values. Due to the large size of the models, it is not possible to fully assess the convergence of the MAEs with respect to the number of relaxed Sb_2_Te_3_ layers and the k-point mesh (this point is further discussed in the Methods section). Nevertheless, this estimate is sufficient for our goal of determining the relative magnitude between the MAE and the AFM coupling strength. It is worth mentioning that the electronic structure of the GNR and the substrate is hardly affected by the direction of magnetization.

The non-spin-polarized and spin-polarized projected density of states (PDOS) for the valence *p* orbitals (more precisely, for the 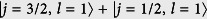 spin-angle functions) of an edge C atom and the nearest neighbour Te atom are shown in Fig. 2(c,d). The interaction-induced splitting of the 2*p* PDOS peaks of the C atom, which reflects the ferromagnetic ordering along the edge, is about 0.3 eV. This value is smaller than the splitting obtained for an unsupported GNR with the same width (0.7 eV). This reduction stems from the non-negligible (albeit small) hybridization between C and the neighbour Te atoms, as shown in the figure. The broadening of the lower peak at the Fermi energy is also due to said hybridization.

**Figure 3:**
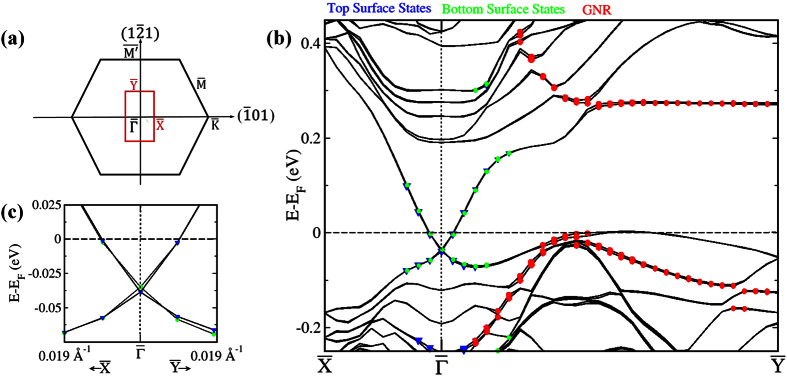
(**a**) The surface Brillouin zone (BZ) of Sb_2_Te_3_(111) (black) and of the orthorhombic supercell employed in our simulations (red). The directions indicated in the figure refer to the rhombohedral lattice. (**b**) Band structure for the H-terminated GNR on Sb_2_Te_3_ along the 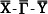 lines of the surface BZ of the supercell for magnetization along x (easy axis). 1) Top and 2) bottom surface state bands and 3) GNR edge state bands are identified by requiring that the sum of the squares of the projections of the states on the orbitals of the atoms of the 1) 5 topmost Sb_2_Te_3_ layers, 2) 5 bottommost layers and 3) edge C row be larger than 0.3. (**c**) Zoomed-in view of the band structure around the 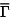 point, which shows more clearly that the top and bottom surface state bands almost coincide.

**Figure 4:**
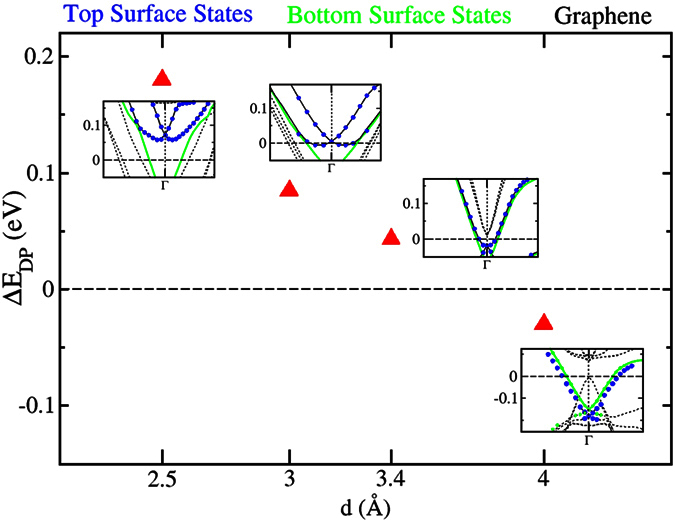
Doping effects in perfect graphene deposited on Sb_2_Te_3_(111). The plot shows the energy difference ΔE_DP_ between the Dirac point of the top surface state of Sb_2_Te_3_ (which interacts with graphene) and the bottom surface state, as a function of the graphene-Sb_2_Te_3_ distance d. Positive (resp. negative) ΔE_DP_ indicate *p*-doping (resp. *n*-doping) of the top surface and, thus, *n*-doping (resp. *p*-doping) of graphene. The insets show the corresponding band structures around 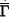 the point (along the directions 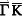 and 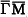). In the insets, energies are in eV and E_F_ is set at 0 eV. Notice that graphene displays a sizable energy gap even at large d, which is induced by the substrate^29^.

The band structure of the system along the 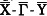 lines of the first Brillouin zone (BZ) of the supercell (with orthorhombic symmetry) is shown in Fig. 3(a) for the case of magnetization along *x* (easy axis). The line 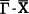 (resp. 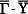) corresponds to the 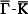 (resp. 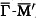) line of the first BZ of the Sb_2_Te_3_(111) surface. Since our supercell contains 3 GNR units along *y*, the portion (2/3π/*a*-π/*a*) of the one-dimensional BZ of the GNR (where *a* is the lattice parameter of graphene) is exactly folded onto the 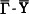 portion of the supercell, see inset of Fig. 3(a). Hence, 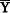 coincides with the boundary of the BZ of the GNR. The bands of the surface states of the top and bottom surface are coloured in green and blue, respectively. There is a tiny shift of the Dirac point of the top surface state to lower energies by about 4 meV. Therefore, doping effects are practically negligible. Notice that a perfect graphene sheet on Sb_2_Te_3_ at the equilibrium distance of 3.4 Å is slightly *n*-doped. However, the type of doping (*n* or *p*) depends on the distance between graphene and the Sb_2_Te_3_. Similarly to what occurs for some metallic substrates^37^, graphene is *n*-doped for small distances and becomes *p*-doped for distances exceeding 3.5 Å, see Fig. 4 (the figure shows data about the doping of the Sb_2_Te_3_ surface, which, obviously, displays the opposite trend). The equilibrium distance turns out to be close to the transition point. In the case of GNRs, the absence of doping can be ascribed to small differences in the chemical interaction with Sb_2_Te_3_, as compared to perfect graphene, which stem from the presence of the edge states.

In principle, the effects of a magnetic perturbation on the surface states of a TI depend on the direction of magnetization. For idealized, rotationally invariant surfaces, a magnetization axis perpendicular to the surface induces a gap in the band structure, whereas an in-plane magnetization does not^16^. However, for surfaces with C_3_ _v_ symmetry like Sb_2_Te_3_(111), in-plane magnetic fields can also open a gap due to hexagonal warping effects^38^ (interestingly, warping terms in hexagonal surfaces had previously been investigated by Henk *et al*.^39^, in the context of the SOC-induced Rashba-splitting of the *L*-surface state of Au(111)^40^,^41^). In our model, no gap is observed, irrespective of the direction of magnetization of the GNR edge states. This behaviour originates from the fact that the exchange interaction between the edge-state electrons and the surface state electrons is weak, owing to the large GNR-substrate distance. Moreover, no evidence for the formation of quasi one-dimensional chiral states at the surface (induced by the AFM configuration of the GNR) is found.

## Discussion and conclusions

We have shown that the chemical interaction between the GNR and the substrate does not upset the mean-field magnetic properties of the GNRs. However, quantum fluctuations (not included in our DFT simulations) will destroy edge magnetism, unless spin-orbit coupling and/or decoherence effects are sufficiently strong. Our simulations indicate that there is an easy axis, which is in-plane and perpendicular to the GNR, and the MAEs are of the order of 0.1-0.2 meV. This energy scale has to be compared with the magnitude of the AFM coupling across the GNR. For this purpose, it is useful to consider a different edge geometry, consisting of zigzag segments separated by steps (chiral GNR), which possesses well localized edge states. This geometry allows for an essentially exact mapping onto a Heisenberg spin ladder with AFM rung coupling J_AFM_ and FM leg coupling J_FM_, as discussed in ref.^11^. Since a) the AFM coupling in free-standing GNRs does not depend strongly on the edge geometry (for fixed GNR width) and b) our results indicate that, for H-terminated GNRs, the reduction in this coupling upon deposition on the Sb_2_Te_3_ substrate is not dramatic (it is a factor of 2 reduction), we can safely conclude that the AFM coupling in deposited GNRs with zigzag and chiral geometry is of the same order of magnitude. We also expect that the strength of the MAE does not depend strongly on the edge geometry, since it is mainly determined by the effective SOC induced by the substrate. We can thus estimate the parameters of the ladder Hamiltonian from our simulations. Since a zigzag segment of length equal to 3 GNR units (corresponding to our supercell) can host a localized state and, thus, an unpaired spin, the anisotropy strength for each edge spin in the effective ladder model is of order 0.1 meV, whereas the AFM exchange rung coupling, proportional to E_FM_-E_AFM_, is of the order of 10 meV. The relatively large value of J_AFM_ is due to the small width *W* of the GNR (*W* = 1.14 nm). According to ref. 42, the spin ladder exhibits a phase transition from a “rung singlet” (corresponding to the entangled singlet state in the GNR) to a “stripe ferromagnetic phase” (corresponding to edge magnetism) as a function of the anisotropy strength. Although the critical anisotropy for a given J_AFM_ also depends on J_FM_ (which is more difficult to determine from DFT simulations, because twice as large supercells along the GNR direction must be employed), it is clear from the phase diagram shown in ref. 42 that, for such small ratio between the anisotropy and J_AFM_, the system is in a singlet state. Since, however, the MAE is expected to be largely width-independent while the J_AFM_ decreases strongly with ribbon width *W*, this material system provides means to study the rung singlet - stripe FM phase transition, which should happen as *W* is increased. Notice that very recent work has shown that effective spin theories are applicable to the zigzag geometry as well^43^. Nonetheless, the long-range nature of the exchange couplings along the leg in the corresponding spin ladder model could lead to differences in the phase diagram with respect to the chiral GNR.

The second important stabilizing mechanism is decoherence, which stems from the coupling of the GNR edge states (the “system”, in the language of open systems) to the surface states of Sb_2_Te_3_, which correspond to the environment. The basic principle of this stabilization can be understood best if decoherence is viewed as the dynamical leakage of quantum information into the environment, so that the environment continuously measures the system. The macroscopic ground state of an isolated GNR is a highly entangled singlet state^11^. The individual electron spins at each edge are aligned with each other and thus give rise to large “superspins”, one at each edge. However, this macroscopic spin points in all directions simultaneously. As soon as such a superposition state is allowed to interact with the environment, information about the alignment of the superspin is acquired by the environment. In the extreme case where the system-environment interaction (which leads to a continuous measurement of the system) dominates over the intrinsic dynamics (which tends to restore the entangled singlet state), this mechanism should lead to a complete collapse of the wave function toward the classical magnetic state, i.e. to edge magnetism. Hence, although this classical state is not intrinsically stable in an isolated GNR, the continuous monitoring by the environment gives rise to an extrinsic stabilization^11^. This is known as the Quantum-Zeno effect^44^: strong decoherence implies that the environment projects the system into the same unstable state over and over again by measuring it^45^,^46^.

We argue that these effects should lead to a quantum-to-classical transition as the ribbon width is increased. Quantitative investigation of these fascinating phenomena requires the derivation of effective low-energy theories (based on our DFT simulations) for the interaction between topological surface states and GNR edge states. The first step of this derivation would be a model of interacting fermions restricted to only the essential degrees of freedom, namely the TI surface states and the graphene π-band. The TI bulk states and the σ band in graphene, which are next-closest to the Fermi level, are both gapped and therefore not a priori important for the low-energy domain. In a second step, this fermionic model should be further reduced to a model of localized spins and their interaction with the TI surface state electrons. Such simplified model should be amenable to many body methods beyond the mean-field approximation. This will be the subject of future work.

In conclusion, our DFT simulations indicate that the chemical interaction between H-terminated GNRs and the Sb_2_Te_3_(111) is weak: as a consequence, there is small structural relaxation of the GNRs. Furthermore, the mean-field magnetic properties of the GNRs are not strongly affected by the presence of the substrate. Exact results about spin ladder Hamiltonians^42^ suggest that the estimated magnetic anisotropy energies (of the order of 0.1 meV) are not sufficiently strong to stabilize edge magnetism against quantum fluctuations, except for very wide GNRs. Nevertheless, decoherence phenomena due to the interaction with the surface states of Sb_2_Te_3_(111) may be effective in restoring classical behaviour and, thus, deserve further investigation.

## Methods

### Computational details

We use the DFT plane-wave package Quantum-Espresso^47^. We employ scalar-relativistic and fully-relativistic norm-conserving pseudopotentials^48^ and local-density-approximation and gradient-corrected Perdew-Burke-Ernzerhof functionals^49^. For the latter, we include the semi-empirical van der Waals corrections by Grimme^50^. The plane-wave cut-off energy is 816 eV (60 Ry). We carry out the geometry optimization without SOC and converge the total energy to 2 × 10^−6 ^eV. Subsequently, we include SOC to determine the electronic and magnetic properties of the relaxed structures. Upon inclusion of SOC, all the components of the forces acting on the atoms change by less than 5 × 10^−3 ^eV Å^−1^. The MAEs are calculated by computing the total energy for different orientations of the magnetization. We employ an orthorhombic supercell of the surface that corresponds to 5 x sqrt(3) of the hexagonal Sb_2_Te_3_(111) unit cell, which has a lattice parameter of 4.26 Å. We consider thick slabs containing 30 layers to model the substrate, separated by a vacuum layer of 17 Å. Thick Sb_2_Te_3_ slabs are required to decouple the surface states on opposite surfaces of the slab^51^. Thinner slabs lead to a spurious gap due to the hybridization between said states. The GNR has a width of 6 graphene units, corresponding to 1.14 nm. The supercell contains 3 GNR units along the *y* direction parallel to the GNR. The GNRs is on the top surface of the slab. In total, the model contains 342 atoms. 1 × 8 × 1 Monkhorst-Pack (MP) meshes^52^ are used to perform the integration over the Brillouin zone. We have also considered a 1 × 4 × 1 MP mesh and found that the two meshes yield the same easy axis. Due to the large size of the models, it is computationally unfeasible to employ denser MP meshes to assess the convergence of the MAEs. All of the atoms of the GNRs and of the four topmost layers of the surface are allowed to relax during structural optimization.

## Additional Information

**How to cite this article**: Zhang, W. *et al*. Electronic and magnetic properties of H-terminated graphene nanoribbons deposited on the topological insulator Sb_2_Te_3_. *Sci. Rep.* **6**, 29009; doi: 10.1038/srep29009 (2016).

## Figures and Tables

**Table 1:** Minimum and maximum distance between the H-terminated GNR and the Sb_2_Te_3_ (111) surface.

	H-terminated GNR	
	Min (Å)	Max (Å)
hollow (a)	3.37	3.49
on-top (b)	3.40	3.58
bridge-1 (c)	3.44	3.56
bridge-2 (d)	3.43	3.57

Distances are in Angstrom. The letters refer to the structures shown in Fig. 1.
